# Emerging therapeutic roles for NAD^+^ metabolism in mitochondrial and age-related disorders

**DOI:** 10.1186/s40169-016-0104-7

**Published:** 2016-07-27

**Authors:** Sarika Srivastava

**Affiliations:** Virginia Tech Carilion Research Institute, 2 Riverside Circle, Roanoke, VA 24016 USA

**Keywords:** Nicotinamide adenine dinucleotide, Oxidative phosphorylation, Mitochondrial disorders, Metabolism, Nicotinamide riboside, Sirtuins, Age-related disorders

## Abstract

Nicotinamide adenine dinucleotide (NAD^+^) is a central metabolic cofactor in eukaryotic cells that plays a critical role in regulating cellular metabolism and energy homeostasis. NAD^+^ in its reduced form (i.e. NADH) serves as the primary electron donor in mitochondrial respiratory chain, which involves adenosine triphosphate production by oxidative phosphorylation. The NAD^+^/NADH ratio also regulates the activity of various metabolic pathway enzymes such as those involved in glycolysis, Kreb’s cycle, and fatty acid oxidation. Intracellular NAD^+^ is synthesized de novo from l-tryptophan, although its main source of synthesis is through salvage pathways from dietary niacin as precursors. NAD^+^ is utilized by various proteins including sirtuins, poly ADP-ribose polymerases (PARPs) and cyclic ADP-ribose synthases. The NAD^+^ pool is thus set by a critical balance between NAD^+^ biosynthetic and NAD^+^ consuming pathways. Raising cellular NAD^+^ content by inducing its biosynthesis or inhibiting the activity of PARP and cADP-ribose synthases via genetic or pharmacological means lead to sirtuins activation. Sirtuins modulate distinct metabolic, energetic and stress response pathways, and through their activation, NAD^+^ directly links the cellular redox state with signaling and transcriptional events. NAD^+^ levels decline with mitochondrial dysfunction and reduced NAD^+^/NADH ratio is implicated in mitochondrial disorders, various age-related pathologies as well as during aging. Here, I will provide an overview of the current knowledge on NAD^+^ metabolism including its biosynthesis, utilization, compartmentalization and role in the regulation of metabolic homoeostasis. I will further discuss how augmenting intracellular NAD^+^ content increases oxidative metabolism to prevent bioenergetic and functional decline in multiple models of mitochondrial diseases and age-related disorders, and how this knowledge could be translated to the clinic for human relevance.

## Introduction

**Fig. 1:**
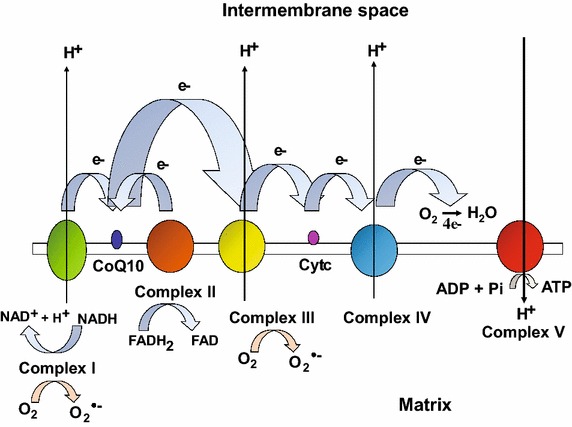
Schematic illustration of mammalian oxidative phosphorylation system. The mammalian OXPHOS comprises of five multimeric enzyme complexes (CI–V). Electrons from reducing equivalents i.e. NADH and FADH_2_ enter mitochondrial electron transport chain (ETC) and reduce complex I and complex II, respectively. An inner membrane electron carrier, coenzyme Q10 or ubiquinone accepts an electron from either complex I or complex II and donates it to complex III. Cytochrome *c*, another electron carrier in the intermembrane space accepts an electron from complex III and donates it to complex IV, which in turn reduces molecular O_2_ to H_2_O. During the electron flow, complex I, III and IV simultaneously pump protons from the matrix towards intermembrane space generating an electrochemical gradient or membrane potential (Ψ_m_) across the inner mitochondrial membrane. The energy in this gradient is harnessed by complex V to generate ATP from ADP and inorganic phosphate (Pi), a phenomenon termed as OXPHOS. Approximately 1–2 % electrons leak from the ETC and reduce O_2_ to superoxide radical (O_2_^•−^) thereby producing reactive oxygen species (ROS)

Mitochondria are highly dynamic intracellular organelles that play crucial roles in energy production, metabolism, intracellular signaling and apoptosis [87, 112]. These organelles are maternally inherited and semiautonomous containing their own DNA (mtDNA) which is a circular double-stranded molecule of ~16.5 kb in mammals encoding 13 polypeptide subunits, 22 transfer RNAs and 2 ribosomal RNAs. The rest of the mitochondrial proteome, consisting of ~1500 additional polypeptides is encoded by the nuclear DNA (nDNA), translated in the cytosol and imported into the organelles by an active process [87]. Mitochondria have the ability to change their morphology, number and function in response to various physiological stimuli (e.g. exercise, diet, temperature, or hormones) and stress [91]. Proper mitochondrial function is therefore critical for the maintenance of metabolic homeostasis and activation of appropriate stress responses. A principal bioenergetic function of mitochondria is to generate adenosine triphosphate (ATP) from nutrient breakdown (e.g. glucose, fatty-acids and amino-acids) through a process termed as oxidative phosphorylation (OXPHOS). This process involves transport of electrons from reduced equivalents [e.g. nicotinamide adenine dinucleotide (NADH) and flavin adenine dinucleotide (FADH_2_)] along the respiratory chain protein complexes (CI-IV) via the electron carriers (e.g. coenzyme Q10 and cytochrome *c*) to the terminal electron acceptor i.e. oxygen (O_2_) which is ultimately reduced to water (Fig. 1) [34]. The electron flow is coupled with the translocation of protons from the matrix to the intermembrane space (via complexes I, III and IV) which in turn generates an electrochemical proton gradient or membrane potential (ΔΨ_m_) across the inner mitochondrial membrane. The energy in this gradient is subsequently harnessed by complex V or ATP synthase to generate ATP from adenosine diphosphate (ADP) and inorganic phosphate (Pi), during when the protons flow back from the intermembrane space to the matrix (Fig. 1) [34]. Under normal conditions ~1 to 2 % of electrons leak from the electron transport chain and reduce O_2_ to superoxide radical (O_2_^•−^) thereby producing reactive oxygen species (ROS), which is detoxified by the action of antioxidant enzymes such as superoxide dismutase, catalase, and glutathione peroxidase [44, 95]. However, when the balance between ROS production overrides the antioxidant capability of the cells, it leads to oxidative stress which is highly damaging to cellular macromolecules (i.e. DNA, lipids and proteins), and is linked to multiple pathologies including neurodegenerative diseases, diabetes, cancer and premature aging [44, 95]. Mitochondrial dysfunction caused by genetic mutations in mtDNA or nDNA encoded OXPHOS proteins affects the electron transport chain (ETC) function and impairs ATP production leading to the onset of mitochondrial diseases wherein the high energy demanding tissues such as brain, heart, retina and skeletal muscle are predominantly affected [34, 93]. Mitochondrial dysfunction is not only a hallmark of mitochondrial disorders, but is also implicated in aging and age-related disorders such as diabetes, obesity, neurodegeneration and cancer.

Sirtuins or silent information regulator 2 (Sir2) proteins are a family of evolutionarily conserved nicotinamide adenine dinucleotide (NAD^+^)-dependent protein deacylases harboring lysine deacetylase, desuccinylase, demalonylase, demyristoylase and depalmitoylase activity [37, 40, 88, 104], or an ADP-ribosyltransferase activity [36, 48]. Mammals contain seven sirtuins (SIRT1–7) that are located in different subcellular compartments i.e. nucleus (SIRT1, SIRT6 and SIRT7), cytosol (SIRT2), and mitochondria (SIRT3, SIRT4 and SIRT5) [49, 114], and are implicated in a wide variety of biological functions including control of cellular metabolism and energy homeostasis, aging and longevity, transcriptional silencing, cell survival, proliferation, differentiation, DNA damage response, stress resistance, and apoptosis [2, 49, 102, 110, 114]. Since sirtuins are NAD^+^-dependent enzymes, the availability of NAD^+^ is one of the key mechanisms that regulate their activity. Sirtuins therefore serve as “metabolic sensors” of the cells as their activity is coupled to changes in the cellular NAD^+^/NADH redox state, which is largely influenced by the availability and breakdown of nutrients [20]. Thus, NAD^+^ is not only a vital cofactor/coenzyme but also a signaling messenger that can modulate cell metabolic and transcriptional responses. Changes in cellular NAD^+^ levels can occur due to modulation of pathways involved in NAD^+^ biosynthesis and consumption. Reduced NAD^+^ levels have been reported in mitochondrial and age-related disorders, and NAD^+^ levels also decline with age [17, 26, 45, 53, 60, 67, 71]. Boosting cellular NAD^+^ levels serves as a powerful means to activate sirtuins, and as a potential therapy for mitochondrial as well as age-related disorders.

## Review

### NAD^+^ biosynthesis, consumption and compartmentalization

**Fig. 2:**
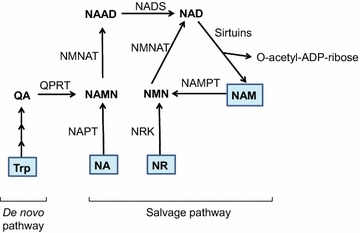
Schematic representation of de novo and salvage pathways for NAD^+^ biosynthesis. In mammals, the de novo biosynthesis starts from l-tryptophan (Trp) which is enzymatically converted in a series of reactions to quinolinic acid (QA). Through quinolinate phosphoribosyltransferase (QPRT) enzyme activity, QA is converted to nicotinic acid mononucleotide (NAMN), which is then converted to nicotinic acid adenine dinucleotide (NAAD) by nicotinamide mononucleotide adenylyltransferase (NMNAT) enzyme. The final step in de novo biosynthesis is the amidation of NAAD by NAD synthase (NADS) which generates NAD^+^. The salvage pathway involves NAD^+^ synthesis from its precursors, i.e. Nicotinic acid (NA), nicotinamide (NAM) or nicotinamide riboside (NR). NA is catalytically converted to NAMN by the action of nicotinic acid phosphoribosyltransferase (NAPT). NAM is converted by nicotinamide phosphoribosyltransferase (NAMPT) to nicotinamide mononucleotide (NMN), which is also the product of phosphorylation of NR by nicotinamide riboside kinase (NRK) enzyme. Finally, NAMN is converted to NAD by the action of NMNAT and NADS enzymes, whereas NMN is converted to NAD by the NMNAT enzyme. Multiple enzymes break-down NAD^+^ to produce NAM and ADP-ribosyl moiety, however only sirtuins are depicted in this figure

The mammalian NAD^+^ biosynthesis occurs via *de novo* and salvage pathways, and involves four major substrates including the essential amino acid l-tryptophan (Trp), nicotinic acid (NA), nicotinamide (NAM), and nicotinamide riboside (NR) [25, 54]. De novo biosynthesis of NAD^+^ starts from dietary Trp which is catalytically converted to N-formylkynurenine by either indoleamine 2,3-dioxygenase (IDO) or tryptophan 2,3-dioxygenase (TDO) and is the first rate limiting step. N-formylkynurenine is then converted by a series of four enzymatic reactions to α-amino-β-carboxymuconate-ε-semialdehyde (ACMS) which is unstable and hence undergoes either complete enzymatic oxidation or non-enzymatic cyclization to quinolinic acid (Fig. 2). The second rate limiting step involves the catalytic conversion of quinolinic acid to nicotinic acid mononucleotide (NAMN) by quinolinate phosphoribosyl transferase (QPRT). Next, NAMN is converted to nicotinic acid adenine dinucleotide (NAAD) by one of the three isoforms of nicotinamide mononucleotide adenylyltransferase (NMNAT) enzyme. The human NMNAT1 is localized in the nucleus, NMNAT2 is found in the Golgi and cytosol, whereas NMNAT3 is localized in both mitochondria and cytosol [13, 54]. The final step of de novo biosynthesis is the amidation of NAAD by NAD synthase (NADS) enzyme (Fig. 2) [25, 54]. The de novo pathway contributes only a minor fraction to the total NAD^+^ pool, however, its importance is stressed by the human disease pellagra which is caused by dietary deficiency of Trp and NAM intermediate, leading to diarrhea, dermatitis, dementia and ultimately death [51]. However, pellagra is easily treated by dietary supplementation of Trp or niacin (i.e. NA, NAM and NR). The primary source of NAD^+^ biosynthesis is the salvage or Preiss-Handler pathway which utilizes dietary niacin as precursors (Fig. 2). The salvage pathway involves catalytic conversion of NA to NAMN by nicotinic acid phosphoribosyltransferase (NAPT), which is subsequently converted to NAD^+^ by the action of NMNAT and NADS enzymes. The NAM and NR are converted to NMN by the action of nicotinamide phosphoribosyltransferase (NAMPT) and nicotinamide riboside kinase (NRK) enzymes respectively. Finally, NMN is enzymatically converted to NAD^+^ by NMNAT (Fig. 2) [25, 54].

The cellular abundance of NAD^+^ is also regulated by its breakdown since NAD^+^ serves as a degradation substrate for multiple enzymes including sirtuins, poly ADP-ribose polymerases (PARPs) and cyclic ADP (cADP) ribose synthases which cleave NAD^+^ to produce NAM and an ADP-ribosyl product [29, 49, 54, 56, 96]. For instance, the deacetylase activity of mammalian sirtuins uses NAD^+^ to cleave the acetyl group from ε–acetyl lysine residues of target proteins to generate NAM and 2′O-acetyl-ADP-ribose. Sirtuins are activated in response to nutrient deprivation or energy deficit which triggers cellular adaptations to improve metabolic efficiency. PARP’s are activated in response to DNA damage (e.g. DNA strand breaks) and genotoxic stress, and use NAD^+^ to catalyze a reaction in which the ADP ribose moiety is transferred to a substrate protein. The cADP-ribose synthases (e.g. CD38 and CD157) use NAD^+^ to generate cADP-ribose which serves as an intracellular second messenger. The members of PARP and cADP-ribose synthase family show increased affinity and lower K_m_ for NAD^+^ compared to sirtuins, indicating that their activation critically impacts intracellular NAD^+^ levels and determines if it reaches a permissive threshold for sirtuin activation [54]. Multiple studies also suggested that PARP activity constitutes the main NAD^+^ catabolic activity, which drives cells to synthesize NAD^+^ from de novo or salvage pathways [14, 98].

Intracellular NAD^+^ has a short half-life, which is estimated to be ~1 to 2 h [38, 84], and is not evenly distributed in subcellular compartments i.e. nucleus, cytosol and mitochondria. Studies report that mitochondrial NAD^+^ levels are higher than in other compartments, for example in mouse skeletal muscles and cardiac myocytes, the mitochondrial NAD^+^ levels were found to be approximately twofold and fourfold higher respectively, than the rest of the cell [1, 77]. Multiple studies indicate that mitochondrial NAD^+^ concentration is ≥250 μM whereas nuclear NAD^+^ concentration is ~70 μM [73, 115], and the nuclear NAD^+^ levels are also lower than the cytosolic NAD^+^ levels [41, 122]. Also, the NAD^+^ pool in each subcellular compartment is partially sequestered from free NAD^+^ by binding to proteins. NAD^+^ cannot diffuse through mitochondrial membranes, therefore changes in cytosolic NAD^+^ levels cannot directly alter the mitochondrial NAD^+^/NADH ratio [9, 79, 109, 115]. Mammalian mitochondria have their own NAD biosynthetic machinery which plays a key role in maintaining mitochondrial NAD pool [13, 115]. However, in yeast NAD is not synthesized in mitochondria but instead transported across the mitochondrial membranes via membrane NAD transporters [106]. A mammalian mitochondrial NAD transporter however has yet to be found. Interestingly, a recent study demonstrated that exogenous NAD can cross the plasma membrane and elevate mitochondrial NAD levels in mammalian cells causing significant enhancement in mitochondrial oxygen consumption and ATP production suggesting the possibility that mitochondrial NAD transport mechanism/s might exist in mammals and that mitochondria can rapidly increase its pyridine nucleotide pool when the cytoplasmic availability of NAD and/or its precursors increases [78].

### NAD^+^ plays a key role in regulating cellular metabolism and energy production

NAD^+^ and its phosphorylated and reduced forms including NADP^+^, NADH, and NADPH are vital in regulating cellular metabolism and energy production. NAD^+^ functions as an oxidoreductase cofactor in a wide range of metabolic reactions and modulates the activity of compartment-specific pathways such as glycolysis in the cytosol, and tri-carboxylic acid (TCA) cycle, OXPHOS, fatty acid and amino acid oxidation in the mitochondria. For instance, NAD^+^ is converted to NADH at the glyceraldehyde 3-phosphate dehydrogenase (GAPDH) step of glycolysis, a pathway that generates pyruvate from glucose [12, 68, 97]. In the mitochondrial compartment, NAD^+^ is converted to NADH at multiple steps in the TCA cycle in which acetyl-coenzyme A is oxidized to carbon dioxide. Mitochondrial NADH is then oxidized by furnishing reducing equivalents to complex I in the ETC through a series of redox reactions that generate ATP from ADP by OXPHOS. The NAD^+^/NADH ratio thus regulates multiple metabolic pathway enzymes including GAPDH, pyruvate dehydrogenase, isocitrate dehydrogenase, α-ketoglutarate dehydrogenase and malate dehydrogenase. In contrast to NAD^+^/NADH, the NADPH/NADP^+^ ratios are maintained high in both cytosol and mitochondrial compartments, to maintain a reducing environment [105]. NADPH plays a key role in reductive biosynthesis and cellular defense against oxidative damage [80]. For instance, NADPH serves as a cofactor for P450 enzymes that detoxify xenobiotics, acts as a terminal reductant for glutathione reductase which maintains reduced glutathione levels during oxidative defense, and also serves as a substrate for NADPH oxidase that generates peroxides for release during oxidative burst processes in the immune system [80].

### Therapeutic potential of NAD^+^ metabolism

Since NAD^+^ is a rate-limiting cofactor for sirtuins, its modulation is emerging as a valuable tool to regulate sirtuin function, and consequently oxidative metabolism. SIRT1 is the most characterized among all sirtuins and is implicated in mitochondrial and metabolic homeostasis [21, 47, 50]. There are multiple targets of SIRT1 including transcriptional co-activators such as the peroxisome proliferator-activated receptor gamma coactivator-1alpha (PGC-1α) and transcription factors such as the forkhead box protein O1 (FOXO1). PGC-1α is the master regulator of mitochondrial biogenesis and function [64, 100, 101], whereas FOXO1 modulates mitochondrial fatty acid metabolism and protects against oxidative stress [108]. SIRT3 is the major mitochondrial deacetylase which targets several proteins involved in fatty acid metabolism, ketogenesis and antioxidant defense [3, 56]. Thus, modulation of NAD^+^ levels has profound effects on oxidative metabolism and mitochondrial function, exerted through a multitude of sirtuin targets, and serve as a promising avenue for the management and treatment of mitochondrial and age-related diseases.

#### Modulation of NAD^+^ levels by physiological processes

**Fig. 3:**
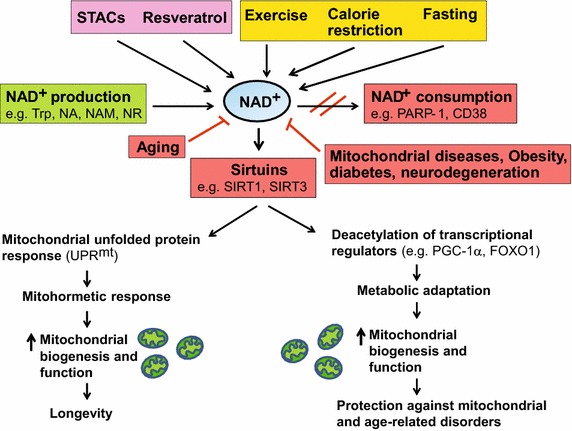
Boosting NAD^+^ levels is beneficial for health and lifespan. NAD^+^ is a rate-limiting cofactor for the enzymatic activity of sirtuins. Boosting intracellular NAD^+^ levels by physiological (e.g. exercise, calorie restriction, fasting) or pharmacological [e.g. resveratrol, sirtuin activating compounds (STACs)] interventions, and inducing NAD^+^ biosynthesis through supplementation with precursors (e.g. NA, NAM, NR) or inhibition of NAD^+^ consuming enzymes (e.g. PARP-1, CD38) leads to activation of sirtuins (e.g. SIRT1, SIRT3). SIRT1 deacetylates and activates transcriptional regulators (e.g. PGC-1α, FOXO1), whereas SIRT3 deacetylates and activates multiple metabolic gene targets (e.g. succinate dehydrogenase, superoxide dismutase 2), which in turn regulate mitochondrial biogenesis and function. Supplementation with NR or PARP inhibitors extends lifespan in worms by inducing the UPR^mt^ stress signaling response via Sir-2.1 activation, which then triggers an adaptive mitohormetic response to stimulate mitochondrial function and biogenesis. Improved mitochondrial function associated with mitohormesis or metabolic adaptation can attenuate the impact of mitochondrial diseases, aging as well as age-related metabolic and neurodegenerative disorders. The physiological and pharmacological interventions that boost NAD^+^ levels are highlighted in *yellow* and *pink* respectively whereas the pathways that produce and consume/decrease NAD^+^ levels are highlighted in *green* and *red* respectively

The intracellular NAD^+^ levels are typically between 0.2 and 0.5 mM in mammalian cells, and change during a number of physiological processes [54]. Since the nucleus, cytosol and mitochondria are equipped with NAD^+^ salvage enzymes, the compartment-specific NAD^+^ production activates distinct sirtuins to trigger the appropriate physiological response. The NAD^+^/NADH levels also vary with the availability of dietary energy and nutrients. For instance, tissue NAD^+^ levels decrease with energy overload such as high-fat diet [23, 118], and display circadian oscillations with a 24 h rhythm in the liver, which is regulated by feeding [4, 74, 83]. During energetic stress such as exercise, calorie restriction (CR) and fasting in mammals, the NAD^+^ levels increase leading to sirtuin activation, which is associated with metabolic and age-related health benefits (Fig. 3) [19, 24, 27, 30]. Decreased sirtuins (e.g. SIRT1 and SIRT3) expression is associated with various age-related pathologies [21, 58, 116, 117, 120, 123] and their overexpression has been reported to enhance overall mitochondrial and metabolic health in age-related disorders as well as mitochondrial diseases [7, 16, 26, 31, 35, 76, 82, 99].

#### Modulation of NAD^+^ levels by pharmacological compounds

Besides physiological processes, NAD^+^ levels can be modulated pharmacologically. Resveratrol—a polyphenolic compound found in red wine has been shown to indirectly stimulate NAD^+^ production by activating the energy sensor AMP-activated protein kinase (AMPK) [22, 42]. Increased NAD^+^ subsequently stimulates SIRT1 activity, which in turn activates PGC-1α and FOXO family of proteins that govern mitochondrial biogenesis and function (Fig. 3) [21, 22]. SIRT1 is also amenable to intervention by small molecules such as SIRT1-activating compounds (STACs) that exert beneficial effects on age-related metabolic abnormalities [21, 71]. NAD^+^ levels can be directly raised by supplying NAD^+^ biosynthetic precursors/intermediates, or by inhibiting NAD^+^ consuming enzymes with specific inhibitors (Fig. 3). For instance, supplementation of NA, NR or NMN compounds increase NAD^+^ levels in both cultured cells and mouse tissues [21, 23, 118]. Because NR can be metabolized both in the nucleus and mitochondria, its supplementation raises the nuclear and mitochondrial NAD^+^ levels, thereby activating nuclear SIRT1 and mitochondrial SIRT3 respectively [21, 23]. Pharmacological activation of NAD^+^ thus stimulates the activity of multiple sirtuin in a compartment-specific manner to exert its beneficial effects on multiple metabolic pathways which is in contrast to STAC’s that specifically stimulate the activity of SIRT1 pathway. Treatment of mice or cultured cells with PARP and CD38 specific inhibitors has also been shown to induce NAD^+^ levels that activate sirtuins [6, 8].

#### Increased NAD^+^ levels protects against mitochondrial and age-related disorders

Mitochondrial disorders represent one of the most common forms of heritable metabolic disease in children [33, 70, 92]. Reduced NAD^+^/NADH ratio is strongly implicated in mitochondrial disorders and, age-related disorders including diabetes, obesity, neurodegeneration and cancer [26, 53, 60, 71]. NAD^+^ levels also decline during aging in multiple models including worms, rodents and human tissue [17, 45, 67, 72]. Increasing evidence suggests that boosting NAD^+^ levels could be clinically beneficial, as it activates the NAD^+^/sirtuin pathway which yields beneficial effects on multiple metabolic pathways.

Pharmacological activation of NAD^+^ production has recently been used to treat mouse models of mitochondrial diseases. For instance, treatment of cytochrome *c* oxidase (COX) deficiency caused by *SURF1*, *SCO2* or *COX15* genetic mutations in mice, with AMPK agonist 5-aminoimidazole-4-carboxamide ribonucleotide (AICAR), partially rescued mitochondrial dysfunction and improved motor performance [111]. These findings could be explained by the fact that AMPK stimulates NAD^+^ production, consequently activating SIRT1 which promotes energy production and homeostasis [21]. Oral administration of NAD^+^ precursor, NR in mitochondrial myopathy mice harboring a pathogenic mutation in the mtDNA helicase—Twinkle, effectively delayed myopathy progression, by increasing mitochondrial biogenesis, preventing mitochondrial ultrastructural abnormalities, mtDNA deletion formation and activating the mitochondrial unfolded protein (UPR^mt^) response [60]. In addition, NR supplementation and reduction of NAD^+^ consumption by a specific PARP inhibitor significantly improved mitochondrial respiratory chain defect and exercise intolerance, in a mouse model of COX deficiency caused by *SCO2* mutation [26].

Besides improving mitochondrial function, boosting NAD^+^ levels with resveratrol, NR or NMN also corrects metabolic disturbances in mice caused by high fat diet [10, 21, 62, 118]. NMN administration ameliorates glucose intolerance and insulin resistance in diet- and age-induced type 2 diabetic mice [82, 118], and rectifies glucose-stimulated insulin secretion and glucose intolerance in NAMPT-deficient animals, by restoring NAD^+^ levels [85]. Interventions using NAD^+^ precursors or PARP inhibitors were also shown to be neuroprotective. For instance treatment with NMN or NR precursors, protected against axonal degeneration and hearing loss in mice [18, 90]. Raised NAD^+^ levels after CR, NAM or NR treatment attenuated increase in β-amyloid content and oxidative damage, preventing cognitive decline and neurodegeneration in rodent models of Alzheimer’s disease [46, 81, 107]. PARP-1 activation also occurs in neurodegenerative DNA repair disorders including xeroderma pigmentosum group A (XPA) and Cockayne syndrome group B (CSB), and treatment with specific PARP inhibitors rescues defective phenotypes in XPA mutant worms and CSB mutant mice respectively [39, 94]. However, PARP-2 deleted mice were glucose intolerant and exhibited pancreatic dysfunction, implying that these results may interfere with other beneficial consequences of PARP inhibition, and hence warrant further investigation on the safe clinical use of these inhibitors [5]. Because PARP inhibitors enhance oxidative metabolism and improve metabolic flexibility, these compounds are being tested in phase III trials as anti-cancer agents [6, 86].

Increasing NAD^+^ levels by treatment with NA and NAM precursors has been shown to inhibit metastasis and breast cancer progression in response to mitochondrial complex I defect in mice [89]. However, reducing NAD^+^ bioavailability is reported to have an antineoplastic effect in various tumor cell types, as cancer cells rely on increased central carbon metabolism and biomass production to sustain an unrestricted growth [28, 103]. The exact role of sirtuins in cancer remains controversial with dichotomous functions being reported, for example multiple studies have shown that SIRT1, SIRT3 and SIRT5 can act as tumor promoters or tumor suppressors under different cellular conditions, tumor stage and tissue of origin [11, 32, 43, 52, 61, 63, 65, 66, 113, 119]. However, SIRT4 is only shown to have a tumor suppressor function [57, 69]. Further research is needed to understand why and how certain sirtuins have both oncogenic or tumor-suppressive roles, and how this dual action may be best exploited for cancer management.

Declining NAD^+^ levels during aging compromise mitochondrial function in multiple model organisms, which can be restored via NAD^+^ precursor supplementation or PARP inhibition. For instance, NMN or NR administration in aged mice or worms respectively, reversed mitochondrial dysfunction by restoring NAD^+^ levels [45, 72, 121]. Moreover, NR administration or PARP inhibition in worms extended lifespan by activating the UPR^mt^ response via *Sir-2.1* (worm *SIRT1* ortholog) and mitonuclear protein imbalance, which in turn induced a mitohormetic response to improve mitochondrial function (Fig. 3) [55, 72]. Inducing UPR^mt^ genes such as *Hsp60* paralogs in *Drosophila* also prevented mitochondrial and age-dependent muscle dysfunction, thereby promoting longevity [75].

## Conclusions and future directions

NAD^+^ has emerged as a vital oxidoreductase cofactor that regulates metabolism, activates sirtuins and maintains mitochondrial function by enhancing oxidative metabolism to promote healthy aging, and can extend lifespan in worms through the UPR^mt^ stress response pathway. The control of mitochondrial and metabolic homeostasis by an evolutionarily conserved NAD^+^/sirtuin pathway has opened an exciting new area of research that holds great clinical potential. Based on the current evidence, both NAD^+^ precursors and PARP inhibitors seem as promising candidates for boosting NAD^+^ levels in cell culture and animal models. However, there are several key questions that remain unanswered. First, whether different pharmacological, genetic and physiological manipulations that boosts NAD^+^ production lead to enhanced activity of all sirtuin enzymes or whether only a few family members are activated, especially considering the fact that some sirtuins may have opposing actions? Second, how sirtuins located in different subcellular compartments differ in their enzyme kinetics towards NAD^+^ availability? Third, what may be the optimal dosages, routes of administration, efficacy and bioavailability of compound drugs that raise intracellular NAD^+^ levels for human application? Future studies that are directed towards understanding these would be highly relevant in designing therapeutic strategies aimed at selective activation of specific sirtuins, and would also aid in translating the results for human clinical application. It is possible that some of the NAD^+^ boosting drugs show adverse side effects in humans which could preclude their use and/or may be acceptable for only those inherited conditions that are highly devastating. It is also important to determine if NR could be valid substitute to avoid undesirable side effects of other NAD^+^ precursors such as NA and NAM, for instance when used as lipid lowering drugs [15, 59]. In addition, future studies are required to examine the UPR^mt^ pathway in vivo in mammalian models to identify key signaling molecules involved in mitochondrial protective mechanisms, which will further advance our understanding of the diseases associated with mitochondrial dysfunction, and will allow discovery of new targets to modulate this pathway. Finally, it remains to be determined whether or not boosting NAD^+^ levels could extend lifespan in higher organisms. Although much remains to be done, based on the steadily growing evidence, the pharmacological modulation of NAD^+^ levels via NAD^+^ precursors and PARP inhibitors appears to be an attractive and valid strategy to enhance oxidative metabolism and mitochondrial biogenesis, and holds a significant therapeutic potential in the clinical management of mitochondrial and age-related disorders.

## Abbreviations

NAD^+^: nicotinamide adenine dinucleotide oxidized
NADH: nicotinamide adenine dinucleotide reduced
NADP^+^: nicotinamide adenine dinucleotide phosphate oxidized
NADPH: nicotinamide adenine dinucleotide phosphate reduced
FADH_2_: flavin adenine dinucleotide reduced
mtDNA: mitochondrial DNA
nDNA: nuclear DNA
OXPHOS: oxidative phosphorylation
ETC: electron transport chain
ATP: adenosine triphosphate
ADP: adenosine diphosphate
ROS: reactive oxygen species
Sir2: silent information regulator 2
Trp: l-tryptophan
NA: nicotinic acid
NAM: nicotinamide
NR: nicotinamide riboside
NMN: nicotinamide mononucleotide
IDO: indoleamine 2,3-dioxygenase
TDO: tryptophan 2,3-dioxygenase
ACMS: α-amino-β-carboxymuconate-ε-semialdehyde
QPRT: quinolinate phosphoribosyl transferase
NAMN: nicotinic acid mononucleotide
NAAD: nicotinic acid adenine dinucleotide
NMNAT: nicotinamide mononucleotide adenylyltransferase
NAPT: nicotinic acid phosphoribosyltransferase
NAMPT: nicotinamide phosphoribosyltransferase
NRK: nicotinamide riboside kinase
PARPs: poly ADP-ribose polymerases
cADP: cyclic adenosine diphosphate
GAPDH: glyceraldehyde 3-phosphate dehydrogenase
CR: calorie restriction
AMPK: adenosine monophosphate-activated protein kinase
PGC-1α: peroxisome proliferator-activated receptor gamma coactivator-1alpha
FOXO1: forkhead box protein O1
STACs: SIRT1 activating compounds
COX: cytochrome *c* oxidase
AICAR: 5-aminoimidazole-4-carboxamide ribonucleotide
UPR^mt^: mitochondrial unfolded protein
XPA: xeroderma pigmentosum group A
CSB: Cockayne syndrome group B
